# Modeling Protein–Glycosaminoglycan Complexes: Does the Size Matter?

**DOI:** 10.1021/acs.jcim.1c00664

**Published:** 2021-09-08

**Authors:** Mateusz Marcisz, Martin Zacharias, Sergey A. Samsonov

**Affiliations:** † Faculty of Chemistry, University of Gdańsk, ul. Wita Stwosza 63, 80-308 Gdańsk, Poland; ‡ Intercollegiate Faculty of Biotechnology of UG and MUG, ul. Abrahama 58, 80-307 Gdańsk, Poland; § Center of Functional Protein Assemblies, Technical University of Munich, Ernst-Otto-Fischer-Str. 8, 85748 Garching, Germany

## Abstract

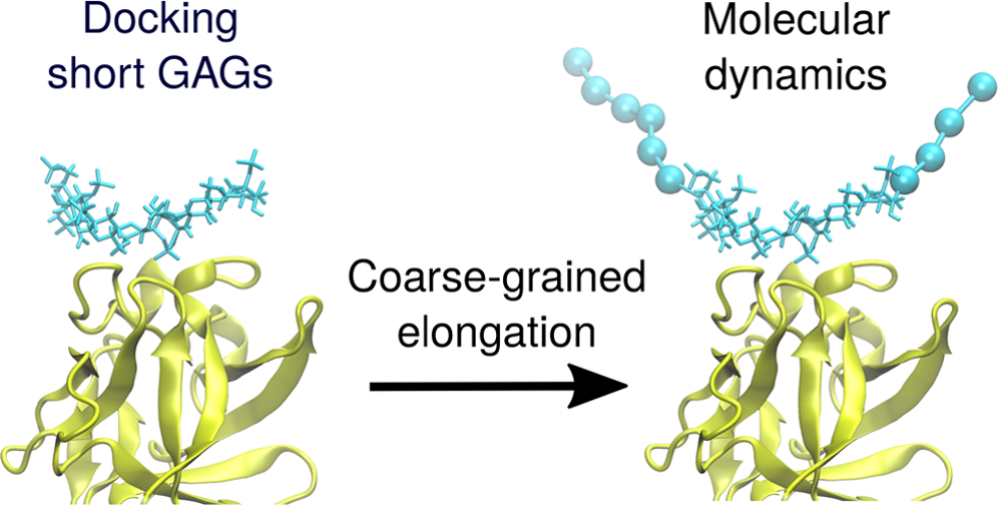

Docking glycosaminoglycans (GAGs) has been challenging because of the complex nature of these long periodic linear and negatively charged polysaccharides. Although standard docking tools like Autodock3 are successful when docking GAGs up to hexameric length, they experience challenges to properly dock longer GAGs. Similar limitations concern other docking approaches typically developed for docking ligands of limited size to proteins. At the same time, most of more advanced docking approaches are challenging for a user who is inexperienced with complex *in silico* methodologies. In this work, we evaluate the binding energies of complexes with different lengths of GAGs using all-atom molecular dynamics simulations. Based on this analysis, we propose a new docking protocol for long GAGs that consists of conventional docking of short GAGs and further elongation with the use of a coarse-grained representation of the GAG parts not being in direct contact with its protein receptor. This method automated by a simple script is straightforward to use within the Autodock3 framework but also useful in combination with other standard docking tools. We believe that this method with some minor case-specific modifications could also be used for docking other linear charged polymers.

## Introduction

Human cells express multiple polymers that display a variety of functions. One particular class of those polymers are glycosaminoglycans (GAGs). They are long periodic linear and negatively charged polysaccharides that by interacting with proteins play an immense role in the extracellular matrix processes. Depending on their sulfation pattern and charge densities, GAGs manifest different conformational and binding properties.^1^ GAGs are built of repeating disaccharide units. Each of them consists of an amino sugar and an uronic acid or galactose.^2^ Depending on the sulfation pattern and monosaccharide composition, GAG disaccharide units can display 408 variants,^3^ of which 202 can be found in mammals.^4,5^ While some of the protein–GAG interactions are specific, most of them are considered as nonspecific and/or electrostatically driven due to the high negative charge of those polysaccharides directly correlating with the binding affinities.^6^ Among many proteins, there are two major protein groups that GAGs can interact with. One of them are growth factors,^7,8^ and the second group are chemokines.^9−11^ In the case of growth factors, GAGs are able to influence the cell signaling and the activity of the proteins by changing their conformation or by oligomerization facilitation of their receptors by binding and clustering multiple fibroblast growth factors (FGFs) at the same time.^12,13^ For example, in the case of vascular endothelial growth factor (VEGF), a key player in cancer, arthritis, angiogenesis, and regenerative processes,^14^ global conformational changes induced by heparin (HP) binding influence its binding capability to its receptor on the cell membrane.^7^ HP and heparan sulfate are also able to bind to transforming growth factor β (TGF -β1),^15,16^ a protein that is responsible for the regulation of the proliferation, adhesion, differentiation, and cell migration.^17^ Depending on the sulfation pattern, hyaluronan derivatives influence TGF-β1 activity and its binding to its receptor.^18,19^ The second mentioned group of the protein that interacts with GAGs are chemokines.^10,20^ This is mostly a proinflammatory group of proteins that belongs to cytokines. They may influence cells in different manners: some of the chemokines can alter metastasis tumor growth and angiogenesis by either promoting or inhibiting it.^21^ GAGs by interacting with IL-8 can alter the ability to activate leukocytes.^22−24^ GAGs can also affect pro-/anti-inflammatory functions of IL-10.^25,26^ It was also shown that HP may interact with CXCL-14,^11^ and by doing that, it increases migratory potential on monocytic THP-1 cells.^27^ Many computational studies on GAGs show their promising potential in the examination of the protein–GAG interactions. The following studies successfully investigated effects of the GAGs binding on a variety of different proteins, such as CXCL-14,^11^ VEGF,^7^ CXCL-8,^9,24,28^ a Proliferation Inducing Ligand (APRIL),^29^ IL-10,^25,26,30^ CXCL-12,^31^ acidic fibroblast growth factor (FGF-1),^32^ or protein–ion–GAG complexes.^33^

Even though computational studies seem to be very successful and helpful in protein–GAG investigations, there are still a lot of challenges that have not been fully overcome yet. One of them is docking long GAG molecules. Usually, GAGs dp4 or 6 (dp stands for degree of polymerization) are used in molecular docking. This is caused by the fact that most of the docking software can handle only a limited number of torsional degrees of freedom for the docked molecules. The number of torsional degrees of freedom is often given by the number of rotatable bonds in the ligand. For example, when using Autodock3, which is the most accurate docking tool for GAG docking,^34^ to dock GAGs of a higher degree of polymerization, a user cannot include all torsional degrees of freedom and needs to manually pick the most relevant ones not to overcome the limit of 33. The more the torsional degrees of freedom are active in a docking procedure, the more accurate docking results should theoretically be possible to obtain. Thus, using very long GAG molecules (e.g., dp10 or higher) heavily hampers docking performance and makes it unfeasible and/or unreliable. However, there are some ways to overcome this issue. In a fragment-based approach, trimeric GAG fragments are docked on the protein’s surface, and afterward, they are assembled based on structural overlaps.^35^ While this method is of great benefit for a number of protein–GAG complexes, it has some flaws, e.g., when GAG is located in a way that some of the oligosaccharide units are in close proximity to the negatively charged amino acid residues (contributing to unfavorable interactions), this method may fail to dock trimeric fragments nearby such residues and thus fail to produce properly docked longer GAG fragments. Perhaps the best method to dock long GAG molecules so far is replica exchange with repulsive scaling method.^36,37^ This method is rather independent of the length of the GAG both in terms of docking predicting power and computational resources requirement (although, this method could demand heavy computational resources—no matter how long the GAG is—depending on the protein size in the complex). This method, while being promising for GAG docking in the vast majority of cases, may experience difficulties to dock GAG molecule into an enzymatic pocket of the protein.^37^ One more argument in disfavor of the above-mentioned specific GAG docking approaches is the fact that they bring in some considerable complexity compared to standard docking methods and may be complicated to handle especially for nonexperts in the molecular modeling and researchers not familiar with the mentioned technical solutions.

Given all that, we aimed to propose a straightforward approach to dock longer GAG molecules without creating unnecessary technical complications while maintaining docking quality. The approach is based on four simple steps: (1) to dock a short (hexameric) GAG; (2) to add more GAG units in the coarse-grained (CG) representation to the previously docked ones manually, e.g., using programs that prepare molecular dynamics (MD) input files like LEaP program from the AMBER suite; (3) to run a molecular dynamics simulation to find an ensemble of GAG conformations for the whole GAG molecule; and (4) to calculate binding free energy. Combining molecular docking with molecular dynamics approaches to predict a complex structure between a receptor and a ligand was previously shown to be a more powerful approach than the usage of the molecular docking alone for other molecular systems.^38,39^ Moreover, the application of molecular dynamics approach allows for the scoring of docking poses with the use of more accurate free energy calculation schemes than it is usually done within molecular docking software and that, in addition, takes into account movements in the molecular system (this aspect is partially or completely neglected in classical docking scoring schemes). In particular, molecular mechanics/Poisson–Boltzmann surface area (MM/PBSA) and its approximation molecular mechanics/generalized Born surface area (MM/GBSA), both based on the use of the implicit solvent model,^40^ showed previously to be able to rank experimental binding poses^41^ and the modeled binding poses^22,42^ for a number of protein–GAG systems in accordance with the experimental data. Apart from this, the per residue free energy decomposition scheme implemented within these methods allows us to dissect individual free energy contributions of the particular residues to the binding affinity allowed and properly rank the effects of point mutations on the binding affinity in the protein–GAG systems.^43,44^ Also, recently, it was shown that the MM/GBSA scoring could be useful in distinguishing a native binding pose from other ones for this type of complexes.^37^

Therefore, our method combining molecular docking, molecular dynamics, and molecular dynamics-based free energy calculation schemes is expected to be more effective than classical docking approaches because of its conceptual superiority, in particular when applied to a GAG ligand that represents numerous challenges for conventional docking protocols.

The study consists of several parts. First, MM/PBSA and MM/GBSA methods to calculate binding free energies are applied to a dataset of protein–GAG experimental structures. The results for all-atom (AA) and coarse-grained (CG) GAGs modeled using previously obtained CG parameters that describe several GAG chemical moieties as different beads^45^ are compared, and the general applicability of these free energy calculation approaches for a CG GAG model is justified. Furthermore, short GAGs from the X-ray structures available for two proteins and GAG docked poses obtained with three peptide receptors are elongated and simulated using a conventional AA approach and the corresponding binding energies are calculated. Then, a new, essentially more simplified, CG model of GAG is introduced. In this model, each GAG monosaccharide unit is represented just by a single pseudoatom. These pseudoatoms are used to substitute the parts of the GAG that are not in contact with the protein/peptide receptor based on the AA simulations. These systems with CG parts are simulated, and the differences between the obtained free binding energies in AA and CG simulations are discussed. Finally, we aimed to propose a model that allows us to calculate free binding energy of a GAG of a given length without simulating the GAG containing an elongated part explicitly using Coulomb and Hückel models of electrostatics. We also attempted to approach the interactions of these GAGs with the protein using only one CG bead to model the elongated part.

We believe that the method for modeling protein complexes with long GAGs proposed in the study with the introduction of some minor changes should also be applicable to most other charged linear polysaccharides or biopolymers like, for example, nucleic acids.

## Materials and Methods

### Comparing the Performances of MM/PBSA and MM/GBSA Free Energy Decomposition Calculations for GAG Ligands in AA and CG Representations Complexed with Proteins

Short (10 ns) MD simulations (see the protocols in the Molecular Dynamics section) were performed for a dataset of nine protein–GAG X-ray structures obtained from the PDB with the following PDB IDs: 1GMN (receptor: NK1; ligand: HP dp5), 1HM2 (receptor: chondroitinase AC lyase; ligand: dermatan sulfate dp4), 1LOH (receptor: hyaluronate lyase; ligand: hyaluronic acid dp6), 1OFM (receptor: chondroitinase B; ligand: chondroitin sulfate-4 dp4), 2D8L (receptor: rhamnogalacturonyl hydrolase; ligand: desulfated chondroitin sulfate dp2), 2NWG (receptor: CXCL-12; ligand: two HP dp2 bound to two different binding sites), 3ANK (receptor: glucuronyl hydrolase mutant D175N; ligand: chondroitin sulfate-6 dp2), 3OGX (receptor: peptidoglycan recognition protein; ligand: HP dp2), 3OJV (receptor: FGF-1 in complex with the ectodomain of FGFR1c; ligand: HP dp6). The dataset included both enzymatic and nonenzymatic proteins previously shown to be characterized by significantly different binding properties^46^ and GAGs of different types and lengths. Two series of the simulations were performed: in the first one, GAGs were described by all-atom model (AA), while in the second one, GAGs were simulated using the coarse-grained representation with the parameters obtained previously (CG).^45^ In this model, specific GAG chemical groups were represented by pseudoatoms, spherical particles described by an integer charge corresponding to the charge of the respective chemical groups and Lennard-Jones parameters. In brief, in this CG representation constructed to be compatible with the AMBER package,^47^ several pseudoatom types were selected to model the pyranose sugar ring (without hydroxyl group substitutes), *N*-acetyl, sulfate, and carboxyl groups, as well as glycosidic oxygen atoms. The bonded parameters (bonds, angles, dihedral angles) were obtained by the Boltzmann inversion approach from the corresponding AA simulations: the distributions of the parameters corresponding to the atomic groups defining pseudoatoms were analyzed, and the corresponding force field parameters fitting the distributions were extracted to define the new atomic types using the AMBER formalism. The charges were assigned empirically, while the Lennard-Jones potential parameters for pseudoatoms were calculated using the potential of mean force approach. Molecular mechanics/Poisson–Boltzmann surface area (MM/PBSA) calculations with default parameters for the whole trajectories of the binding free energies as well as per residue decomposition analysis was performed for the whole obtained trajectories.

Furthermore, the dynamic molecular docking approach (DMD)^48^ was applied to the structures obtained from the PDB with the following PDB IDs: 1BFB (receptor: FGF-1; ligand: HP dp4), 1BFC (receptor: FGF-1; ligand: HP dp6), 2NWG (receptor: CXCL-12; ligand: HP dp2), 3C9E (receptor: cathepsin L; ligand: chondroitin sulfate-4 dp6), 2JCQ (receptor: CD44; ligand: hyaluronic acid dp7). In these simulations, the GAG molecules were treated as CG, and the obtained results were compared with the AA DMD results for the same protein–GAG complexes from the original DMD work.^48^ In brief, the DMD approach uses targeted molecular dynamics protocol to dock a GAG ligand to a protein receptor by applying an additional potential to move a ligand from a distant starting position (beyond the cutoff of nonbonded interactions) to the predefined binding site on the receptor surface. DMD performance was compared for AA and CG ligand models of GAGs. The details for the applied protocols can be found in the original DMD work. The following parameters were included for this comparative analysis: RMSatd_top_: structural difference between the best scored docked structure and the corresponding experimental structure; RMSatd_best_: structural difference between the docked structure, which is the most similar structure to the corresponding experimental structure and the corresponding experimental structure; Rank_best_; rank of the docked structure, which is the most similar structure to the corresponding experimental structure; RMSatd: mean structural difference between all docked structures and the corresponding experimental structure; RMSatd_top cluster_: mean structural difference between all docked structures from the cluster of solutions with the highest scores and the corresponding experimental structure; *r*(Δ*G*_total_ ∼ RMSatd): Pearson correlation coefficient for total free binding energy and RMSatd of all docked structures; *r*(Δ*G*_elect_ ∼ RMSatd): Pearson correlation coefficient for *in vacuo* electrostatic free binding energy component and RMSatd of all docked structures; number of correctly predicted residues; number of correctly charged predicted residues; and number of correctly predicted uncharged polar residues were referenced to the 10 protein residues with the highest impacts on binding according to the per residue decomposition for the corresponding X-ray structures.

### Structures Used in the GAG Elongation Analysis

#### Protein Structures

The following X-ray experimental structures from PDB was used in this work: 1AMX, 2AXM (FGF-1 with HP dp4 and dp6, respectively, monomeric form was used; dp stands for degree of polymerization),^49^ 1BFB, 1BFC (FGF-2 with HP dp4 and dp6, respectively).^13^

#### Peptide Structures

The structure of the N-terminal fragment of the APRIL protein (ALA-VAL-LEU-THR-GLN-LYS-GLN-LYS-LYS-GLN) was adopted from Marcisz et al.^29^ The structures of both peptides GLY-LYS-GLY-LYS-GLY and LYS-GLY-GLY-GLY-LYS (called InLYS and OutLYS, respectively) were constructed using xleap tool from AMBER suite.^47^ Afterward, in the case of both peptides, 100 ns MD runs (described in the Molecular Dynamics section) were performed in AMBER to obtain most probable peptide conformations. The APRIL-derived peptide was chosen to represent a naturally existing GAG binding epitope, while InLYS and OutLYS, peptides were artificially constructed as short positively charged model peptides with the difference in the sequential and spatial distance between the GAG binding positively charged LYS side chains.

#### GAG Structures

All of the full-atom GAG structures—HP dp4 and dp6, dp10, dp16—were constructed from the building blocks of the sulfated GAG monomeric units’ libraries^22^ compatible with AMBER16 package. ^47^GLYCAM06 force field^50^ and literature data^51^ were the sources of GAGs’ charges.

### Molecular Docking

Since there are no available experimental structures of the peptides with HP, for all three peptides, Autodock3^52^ was used as it was previously described to yield the best results for protein–GAG complexes.^34,41^ Entire peptides were covered using maximum gridbox size (126 Å × 126 Å × 126 Å) with a 0.375 Å grid step. The size of 300 for the initial population and 10^5^ generations for termination conditions were chosen. A total of 1000 independent runs with Lamarckian genetic algorithm was used, and 9995 × 10^5^ energy evaluations were performed. DBSCAN algorithm^53^ was used for clustering. RMSatd metric was used for clustering, which accounts for equivalence of the atoms of the same atomic type. This metric was reported to be more appropriate for GAG docking than classical root-mean-square deviation (RMSD) for periodic ligands.^48^

### Coarse-Grained Model Parameters for a Docked GAG Oligomer Elongation

Obtained in this work, CG parameters compatible with AMBER format were obtained by the Boltzmann inversion approach and saved as the Parameter modification file (file.frcmod, see the Supporting Information). These parameters are described in the Results and Discussion section. These new parameters were obtained to be used for the MD simulations of the docked GAG in the AA representation that was further elongated by CG units. Each monomeric unit was represented by a single pseudoatom.

### Molecular Dynamics

Experimental structures of protein–GAG, the docked structures of peptide–GAG complexes, and the corresponding structures with elongated GAGs were further analyzed by the MD approach. All of the MD simulations were performed using AMBER16 software package.^47^ The ff14SB force field parameters were used for the protein and peptide molecules, while GLYCAM06j-1 parameters were used for GAGs. 8 Å water layer from solute to box’s bordes in shape of truncated octahedron was used to solvate complexes. Even in the case of HP dp16, this size of the layer was verified to be sufficient enabling the whole GAG molecule to always remain in the periodic box unit during the MD simulation. Na^+^/Cl^–^ counterions were used to neutralize the net charge of the system. Preceding the production MD runs, energy minimization was made. A total of 500 steepest descent cycles and 10^3^ conjugate gradient cycles with 100 kcal mol^–1^ Å^–2^ harmonic force restraint were performed. It continued with 3 × 10^3^ steepest descent cycles and 3 × 10^3^ conjugate gradient cycles without any restraints and followed by heating up the system to 300 K for 10 ps with harmonic force restraints of 100 kcal mol^–1^ Å^–2^ with the Langevin thermostat (γ = 5 ps^–1^). Afterward, the system was equilibrated at 300 K and 10^5^ Pa in isothermal isobaric ensemble for 500 ps with the Langevin thermostat (γ = 5 ps^–1^) and Berendsen barostat (taup = 1 ps). Then, the actual MD runs were carried out using the same isothermal isobaric ensemble for 100 ns. Particle mesh Ewald method for treating electrostatics and SHAKE algorithm for all of the covalent bonds containing hydrogen atoms were implemented in the MD simulations. For both AA and CG simulations, the integration step of 2 fs was used.

Although we used short 10 ns MD simulations for a dataset of the experimental structures with short GAGs in the first part of our work (see the Comparing the Performances of MM/PBSA and MM/GBSA Free Energy Decomposition Calculations for GAG Ligands in AA and CG Representations Complexed with Proteins section), here we used 100 ns for all modeled complexes with elongated GAGs with the purpose of obtaining more proper sampling of the GAG conformational space when starting from a docked/modeled structures that cannot be verified by experimental data.

### Binding Free Energy Calculations

For the free energy and per residue energy decomposition calculations, MM/GBSA (molecular mechanics generalized Born surface area) model igb = 2^54^ from AMBER16 was used with default parameters on the whole trajectories (100 ns) obtained from MD simulations. Linear interaction energy (LIE) analysis was performed with a dielectric constant of 80 and noncalibrated weights (both α and β were set to 1), performed by CPPTRAJ scripts on the same frames as the MM/GBSA.

## Results and Discussion

### MM/PBSA Calculations for Protein–GAG Complexes: AA vs CG Representation of a GAG

Prior to analyzing the elongated AA-GAG ligands bound to the proteins with the CG part, which represents the focus of this study, we performed MM/PBSA calculations of the binding free energies for nine nonredundant representative protein–GAG complexes where the full GAGs are modeled by with the AA and CG approaches. The aim of these calculations was to find out if the MM/PBSA method yields the results for a system containing a CG part that are in agreement with the data obtained for a conventional AA system. The CG parameters used to obtain the data provided in this subsection were described in detail in the work of Samsonov et al.^45^ The data are summarized in Table 1. Pearson and Spearman correlations for Δ*G*_elect_, Δ*G*_vdW_, and Δ*G*_total_ are 0.997, 0.645, and 0.920; and 0.988, 0.503, and 0.758, respectively, suggesting that CG approximation, as it would be expected, affects van der Waals energy components but retains a very similar description of the systems in terms of the electrostatics. Since the electrostatic interactions are dominating in the protein–GAG systems, the total binding free energies were very similar as well. This suggests that the introduction of the CG part of a GAG that only interacts with the protein receptor via electrostatic interactions could be properly described by the MM/PBSA or MM/GBSA calculations compatibly with similar calculations for AA GAG representation. However, this conclusion should be taken with care: even if the effects of van der Waals description inaccuracies originated from the CG model do not directly affect electrostatic component of binding, they affect the general flexibility of the bound molecule. CG GAGs were shown to be indeed in general less flexible than the AA ones in the original work on this CG model.^45^ Therefore, the introduction of the CG description affects GAG conformational space and, as a consequence, the whole structural organization of the bound GAG. This, in turn, results in the indirect effect of the modified van der Waals interactions on the electrostatics of the system influencing the binding affinity.

**Table 1:** MM/PBSA Free Binding Energy Analysis for Protein–GAG Complexes: Comparison of AA and CG GAG Representations^a^

	AA GAG model			CG GAG model		
PDB ID	Δ*G*_elect_ (kcal mol^–1^)	Δ*G*_vdW_ (kcal mol^–1^)	Δ*G*_total_ (kcal mol^–1^)	Δ*G*_elect_ (kcal mol^–1^)	Δ*G*_vdW_ (kcal mol^–1^)	Δ*G*_total_ (kcal mol^–1^)
1GMN	–3354.6 ± 80.1	–42.2 ± 4.8	–92.6 ± 7.8	–3625.8 ± 84.3	–53.2 ± 4.7	–98.8 ± 9.0
1HM2	–458.6 ± 46.3	–47.2 ± 6.5	–22.4 ± 10.5	–539.4 ± 9.8	–61.7 ± 9.8	–80.6 ± 14.8
1LOH	–42.5 ± 34.1	–76.5 ± 6.6	–55.6 ± 11.7	–103.3 ± 6.3	–31.8 ± 34.4	–109.8 ± 9.7
1OFM	–746.5 ± 52.8	–27.7 ± 3.8	–42.1 ± 9.9	–767.2 ± 47.9	–27.2 ± 6.2	–50.9 ± 12.3
2D8L	–30.7 ± 21.2	–25.3 ± 3.9	–5.5 ± 9.9	–44.9 ± 35.7	–35.1 ± 5.3	–40.2 ± 10.7
2NWG	–1737.9 ± 102.4	–22.4 ± 5.2	–55.5 ± 18.5	–2334.1 ± 126.6	–33.5 ± 8.1	–94.4 ± 19.6
	–1096.7 ± 57.7	–21.5 ± 2.9	–25.1 ± 6.6	–1158.5 ± 106.6	–35.4 ± 7.3	–57.9 ± 12.8
3ANK	3.9 ± 45.1	–41.0 ± 4.5	–22.1 ± 7.0	–83.7 ± 48.4	–52.9 ± 6.5	–88.6 ± 16.9
3OGX	–1235.8 ± 35.7	–53.9 ± 4.3	–51.6 ± 8.7	–1351.7 ± 53.6	–54.3 ± 5.0	–57.3 ± 11.1
3OJV	–5701.5 ± 175.0	–86.0 ± 6.6	–194.9 ± 14.5	–5978.7 ± 148.1	–88.4 ± 6.2	–233.2 ± 15.6

a Δ*G*_elect_, Δ*G*_vdW_, and Δ*G*_total_ are in vacuo electrostatic, van der Waals, and total MM/PBSA binding free energy values, respectively.

The relative mean differences between AA and CG absolute energy values (normalized by the AA corresponding values) are 30, 5, and 18% for Δ*G*_elect_, Δ*G*_vdW_, and Δ*G*_total_, respectively (clear outlier 3ANK is excluded). For all components, the values obtained with CG approach are overestimated in comparison to the ones from the AA approach. Per-residue free energy decomposition also shows systematic agreement for the AA and CG approaches when analyzing the individual impacts of the protein residues (Table S1). At the same time, there are no correlations in the per residue values obtained for the GAG residues. Furthermore, we compared the performances of the DMD docking approach using both AA and CG GAG representations (Table S2). The results obtained for the CG GAG model are slightly worse but, in general, quite similar to the ones obtained for the AA GAG model in the original DMD study.^45^ All of these analyses suggest that the CG description of a GAG molecule complexed with a protein is consistent with the AA representation in terms of application of the MM/PBSA. This served as a premise for our further step in this study: in particular, for the proposition of even a more simplistic CG model for a GAG part that does not establish direct contact with a protein receptor. In this model, the interactions between this CG part of a GAG and the protein could be described as purely electrostatics-driven.

### All-Atom Simulations

To obtain the reference data for the CG model, development and testing AA MD simulations were performed. For this, the available experimental structures of FGF-1 (PDB ID: 1AXM, 2AMX) and FGF-2 (1BFB, 1BFC) with HP dp4 and dp6 were used. These complexes could be successfully obtained by many conventional docking programs including AD3 (RMSD ∼2.5 to 3.5 Å for the best scored docked poses).^34,41^ Since the experimental structures with the peptides are not available, HP dp4 and dp6 were docked to all of the peptides: N-terminal part of the APRIL protein, InLYS, and OutLYS (all targets described in the Materials and Methods section). It is important to mention that in this work, we did not aim to improve the docking quality for short GAGs but to estimate the effect of the GAG elongation and to understand if this elongation could be described properly using a mixed AA/CG GAG model. AA representation of GAGs was used as a reference for our analysis.

Since MM/PBSA and MM/GBSA approaches yielded essential correlation in protein–GAG systems (see an example in Figure S1), we further used only the MM/GBSA approach for these longer simulations since this approach is significantly faster.

We clearly observe that longer GAGs bind stronger independently of the analyzed system and the type of the receptor (protein or peptide) (Table 2). This is an expected net effect of the electrostatic interactions that become stronger with the increase of the GAG negative charge upon its elongation. Since the net charge of a GAG binding site on the protein/peptide surface always corresponds to the extent of the positive electrostatic potential,^41^ an elongation of any GAG ligand bound to any of its receptors would render the interactions stronger. Although the specific binding unit of GAGs is relatively short according to the available PDB structures of protein–GAG complexes,^41^ natural GAGs in the extracellular matrix are very long, reaching molecular weights up to over 100 kDa,^5^ rendering the energetic effect originating in a GAG long chain to be important to take into account when the corresponding modeling is performed. Except the 2AXM, the difference between dp6 and dp16 in terms of binding free energy was 20% or higher (on average 24%). One more highlight of this comparison is that the energy discrepancy between dp6 and dp10 was 2 times higher than that between dp10 and dp16 despite addition of more sugar ring units in the case of dp10 to dp16 elongation. A very large increase in terms of binding strength was observed upon the elongation from dp4 to dp6, indicating that experiments with dp4 GAGs may strongly underestimate the binding strength of longer GAGs. Taken into account how often dp4/dp6 GAGs are used as models in computational studies, it is worth checking and rethinking those standards prior to applying dp4-based protocols to any new system.

**Table 2:** MM/GBSA Analysis of Binding HP of Different Lengths

	OutLys (kcal mol^–1^)	InLys (kcal mol^–1^)	APRIL peptide (kcal mol^–1^)	2AXM (kcal mol^–1^)	1BFC (kcal mol^–1^)
dp4	–24.2	–23.4	–25.6	–71.9	–65.7
dp6	–31.2	–27.6/19.6^a^	–27.1	–84.8	–112.1
dp10	–35.9	–33.6	–42.8	–91.3	–126.2
dp16	–39.2	–36.9	–51.4	–86.6	–144.7

a In the case of one MD simulation, dissociation was observed. The first value indicates energies w/o mention of MD run, and the second value indicates those when taking it into account.

### CG Parameters Obtained from All-Atom MD Simulations

The new parameters described below were obtained from the AA MD simulations to be used for the CG elongation of the docked GAG in the AA representation as described in the following subsections. This new model was particularly developed for the purpose of elongating those parts of bound GAG chains that do not establish direct contact with the protein these GAGs are interacting with, and, therefore, it is thought to account only for electrostatics. Containing a single new atomic type corresponding to a whole GAG monomeric unit, this model is conceptually different and much more simple than the old one.^45^ It is completely nonspecific for any chemical modifications of GAG residues since it is constructed to account primarily for electrostatic interactions and could be used for all negatively charged monosaccharide residues allowing for a straightforward modification of the residue point charge when needed. This is not the case for the old model that, on the contrary, was developed to consider specific electrostatic and van der Waals interactions for particular monosaccharide units. In terms of the required computational expenses, MD simulations with the new model would be faster if only a CG GAG would be simulated. However, in the presence of a protein, an AA-GAG part, and explicit water molecules, the benefit in terms of the computational time reduction is rather negligible.

The new Z1 atomic type constructed corresponds to a CG pseudoatom describing a complete residue unit (monosaccharide unit with the charge of −2) and, therefore, glycosidic linkages are omitted between monosaccharide units in this CG model.

#### Bonded Parameters

Bonded parameters (bonds, angles, and dihedral angles) were obtained from the AA MD simulations. For the calculations of equilibrium values and harmonic constants for bonds, angles, and dihedral angles (Tables 3–5), the Boltzmann inversion approach was used.^55^ In the case of dihedral angles (Table 5), periodicity was set to 1 or 3 depending on the number of maxima/minima of the potential per 360°, and the amplitude was obtained as the difference between the global minimum and the highest energetical barrier between global and local minima. In the case of artifacts observed during simulations, particular parameters were manually refined.

**Table 3:** Z1 Pseudoatom Bond Parameters Compatible with the AMBER Package

covalent bond parameters		
atoms RK	(kcal mol^–1^ Å^–2^)^a^	REQ (Å)^b^
Z1-Z1	120	5.2
Z1-Cg	120	5.2
Os-Z1	120	2.8

a Force constant.

b Equilibrium bond length.

**Table 4:** Z1 Pseudoatom Angle Parameters Compatible with the AMBER Package

angle parameters		
atoms in the angle	TK (kcal mol^–1^ rad^–2^)^a^	TEQ (deg)^b^
Z1-Z1-Z1	100	160
Z1-Z1-Cg	100	160
Z1-Cg-H2	70	108.5
Z1-Cg-Cg	70	108.5
Z1-Cg-Os	60	110
Cg-Os-Z1	100	160
Os-Z1-Z1	100	160

a Force constant.

b Equilibrium angle value.

**Table 5:** Z1 Pseudoatom Dihedral Angle Parameters Compatible with the AMBER Package

dihedral angle parameters				
atoms in the dihedral angle	IDIVF^a^	PK (kcal mol^–1^)^b^	phase (deg)^c^	PN^d^
Z1-Z1-Z1-Z1	1	1	0	1
Z1-Z1-Z1-Cg	1	1	0	1
Z1-Z1-Cg-Cg	1	0.16	0	3
Z1-Cg-Cg-H1	1	0.16	0	3
Z1-Cg-Cg-H2	1	0.16	0	3
Z1-Z1-Cg-H2	1	0.16	0	3
Z1-Z1-Cg-Os	1	0.16	0	3
Z1-Cg-Cg-Ng	1	–1.3	0	1
Z1-Cg-Cg-Cg	1	–0.27	0	1
Z1-Cg-Os-Cg	1	–0.27	0	1
Cg-Cg-Os-Z1	1	0.16	0	3
Cg-Os-Z1-Z1	1	0.16	0	3
H1-Cg-Os-Z1	1	0.27	0	3
Z1-Cg-Cg-Os	1	0.16	0	3
Os-Z1-Z1-Z1	1	0.16	0	3

a Factor by which the torsional barrier is divided.

b Barrier height divided by a factor of 2.

c Phase shift angle in the torsional function.

d Periodicity of the torsional barrier.

#### Nonbonded Parameters (Charges, Lennard-Jones Parameters)

The charge of the pseudoatom of the monomeric unit of the HP was set accordingly to the number of sulfate and carboxyl groups, which is −1 per group in the unit. In the case of Lennard-Jones parameters, the RvdW (van der Waals radius) and EDEP (energy well depth) values were empirically assigned to the doubled and equal values obtained for the internal pyranose ring in our previous CG model of GAGs, respectively (Table 6).^45^

**Table 6:** Z1 Pseudoatom Lennard-Jones Parameters Compatible with the AMBER Package

basic information		Lennard-Jones parameters	
CG pseudoatom	mass (au)	RvdW^a^ (Å)	EDEP^b^ (kcal mol^–1^)
Z1	225	4	3.4

a van der Waals radius.

b Energy well depth.

### Mixed AA/CG Simulations: CG Elongation of a GAG

**Figure 1:**
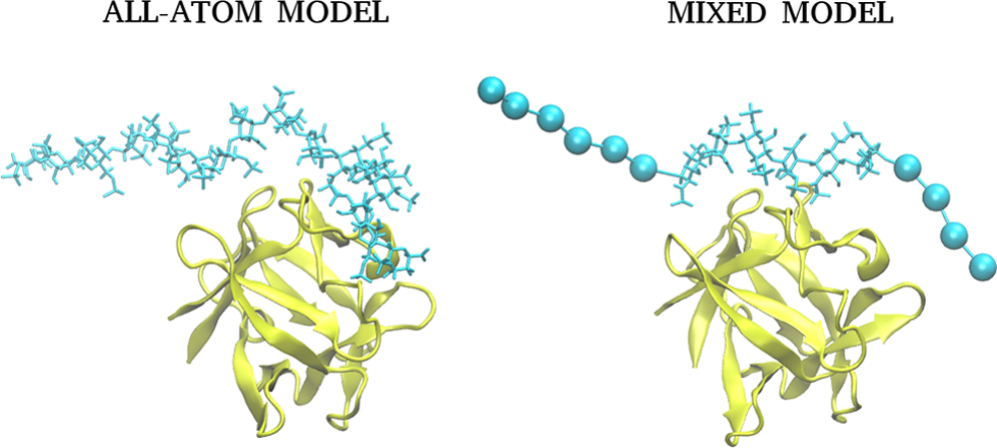
Graphical representation of all-atom (left) and mixed (right) model of dp16 heparin in complex with FGF-2. Protein is in cartoon representation (yellow); all-atom and CG GAGs are in licorice and van der Waals sphere representation, respectively (cyan).

**Figure 2:**
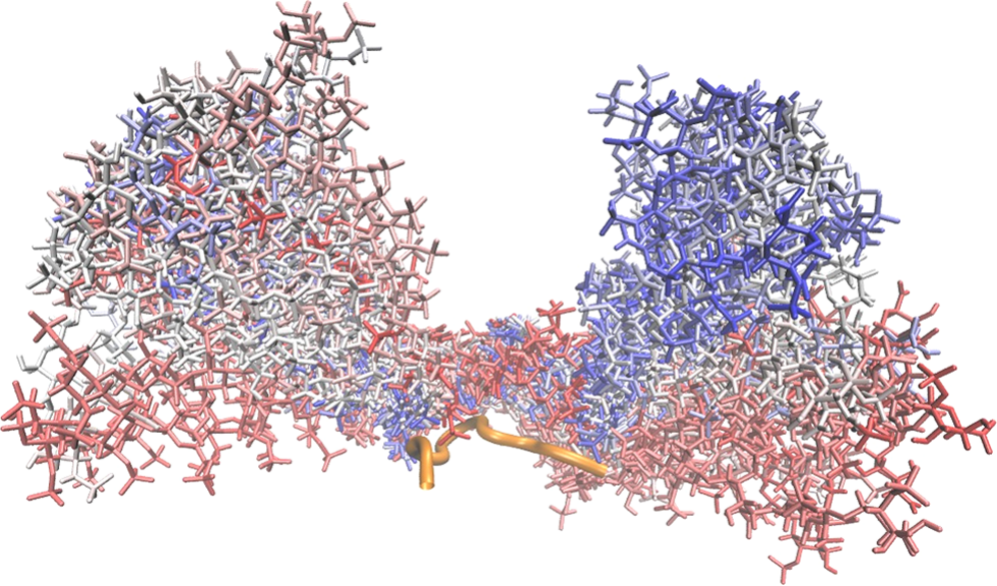
Graphical representation of the MD run of complex of APRIL peptide (orange cartoon) with HP dp16 (licorice). The color scheme from red to blue indicates heparin conformations ranging from the beginning to the end of the MD simulation.

To evaluate our CG model (Figure 1) of the HP, MD simulations with CG atoms were performed and compared to all-atom MD simulations. In AA runs, we observed that the core of GAG—the part that is especially the closest to the binding side of the protein/peptide—is in the closest proximity of the protein and barely moved. In contrast, it is the lateral parts of the GAGs that tend to move freely (Figure 2). It suggests that interactions between those parts and the protein are even less specific and thus almost purely electrostatics-driven. Therefore, we believe that replacing lateral parts of the GAGs with CG model units should not substantially affect the nature of the interactions established between the analyzed molecules.

First, we compared the convergence of MD simulations for the AA and CG approaches in terms of the structural flexibility and energetics (Figures S2 and S3, respectively). In most of the cases, the convergence in terms of RMSD was observed already after 20 ns. Clearly, the flexibility of the AA GAGs is significantly higher than in the mixed AA/CG model. For MM/GBSA binding free energy, the converge is already reached after 10 ns of the simulation, and there are slightly higher variations of the energy observed for the AA simulation, while there are no differences in the time needed for the convergence. The trends of the convergence observed here should not be expected to be the same for other protein–GAG or peptide–GAG complexes. Indeed, in other systems, MD simulations may take longer or shorter to converge. Nevertheless, the goal of the MD simulations performed in this study is not to reach a convergence but to show that the transition from AA to CG representation of the GAG part does not substantially affect the results of the free energy calculations in the same system.

Starting positions of the molecules from all-atom simulations were taken. Original dp6 part of the HP was not modified, and only atoms that were manually added to build dp16 were replaced with CG pseudoatoms for HP rings. Additionally, the user can use the script (Supporting Information) for automatic addition of pseudoatoms. Then, MD simulations with a GAG represented as AA in the binding core and as CG in its lateral parts were performed, and the results obtained from MM/GBSA energy analysis from mixed model simulations of dp16 HP are listed in Table 7. Average difference obtained from energy analysis of mixed CG/AA model compared to the AA model was 5.6%. Compared to the difference that is a consequence of using shorter GAGs, which is on average 24% (dp6) and 39% (dp4) underestimation of the value, it is a substantial improvement. In the case of the mixed model, most of the values were also underestimated (compared to the AA model): 7% for the N-terminal fragment of APRIL, 3% for the FGF-2 and OutLYS peptide, and 1% for the InLYS peptide. However, the binding free energy calculations showed 14% overestimation in the case of the FGF-1/HP complex. Additional energy analysis was performed in the form of LIE calculations and is described in the Supporting Information (Table S3).

**Table 7:** MM/GBSA Energy Analysis from Mixed Model Simulations of dp16 HP

model	description	OutLys (kcal mol^–1^)	InLys (kcal mol^–1^)	APRIL peptide (kcal mol^–1^)	2AXM (kcal mol^–1^)	1BFC (kcal mol^–1^)
AA	AA residues	–39.2	–36.9	–51.4	–86.6	–144.7
AA/CG	elongated fragments of the GAG replaced with CG residues	–37.9	–36.8	–47.8	–98.3	–140.2
	AA residues replaced with CG residues based on decomposed energy values	–46.4	–30.8	–51.0	–90.1	–130.5

**Figure 3:**
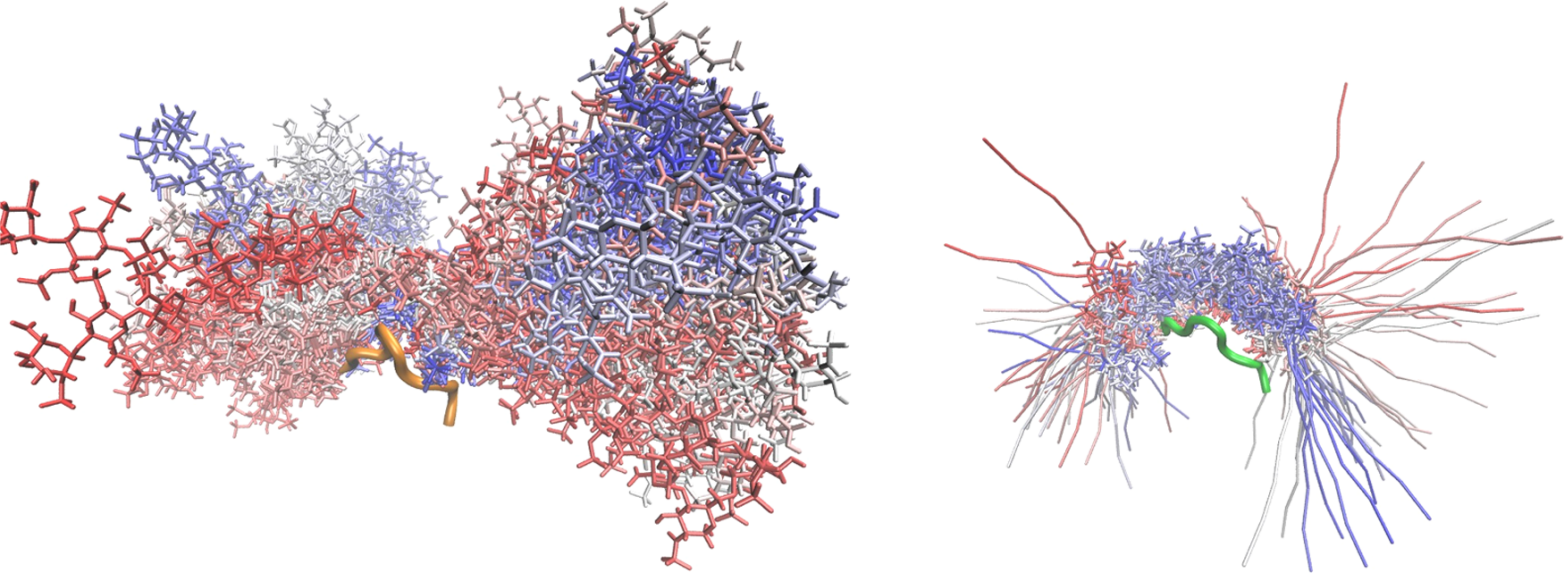
Graphical representation of the MD run of complexes of APRIL peptide (cartoon) with all-atom (left, orange) and mixed model (right, green) HP dp16 (licorice). The color scheme from red to blue indicates heparin conformations ranging from the beginning to the end of the MD simulation.

During MD runs of both AA and mixed AA/CG models, we observed similar motions of the GAGs molecules with respect to the protein/peptide, which suggests that the used CG model also properly reflects the dynamics of the system (Figure 3).

### Mixed All-Atom/Coarse-Grained Simulations Based on Per-Residue Energy Analysis

The division of the modeled GAG chain into AA and CG parts for the further MD analysis could be done by analyzing the free energy properties of the binding poses instead of using visual inspection of AA MD followed by the manual selection of the residues to substitute. For this, we performed per-residue energy analysis of the complexes from AA MD simulations. This procedure allows us to define the particular contributions of the individual GAG units to binding a protein or a peptide. Then, only the residues with “weak” contributions to the binding energies were selected and further modeled by the CG approach. The threshold was set to −0.5 kcal mol^–1^, and any residue with energy value less favorable than this value was replaced. The idea behind such a procedure to substitute only the monosaccharide units with less substantial contributions in terms of binding energy is related to our goal to use the CG model for residues that are further away from the binding region and so less affecting the binding. Interestingly, the obtained error was higher (on average 10% of free energy difference compared to the AA simulation) when the residues were picked based on per-residue free energy decomposition than when the elongation was completed independently of such calculations (Table 7).

### Energy Prediction for GAG Elongation

Furthermore, we aimed to extrapolate binding energies obtained from the analysis of the dp6 GAG to calculate them for the elongated GAG molecules without performing any further MD simulations. First, we proposed an equation based on Coulomb’s law to calculate the factor (depicted as *W* factor) that would allow us to obtain the binding energy of the complex containing GAGs of any length. Such an approach assumes that only electrostatic interactions are substantial for the added GAG part. We also proposed a script (see the Supporting Information) that would automatically calculate the binding energy of the elongated fragment of the GAG when given two files (pdb file of a bound GAG molecule and a receptor) and predefined *W* factor.

To calculate the *W* factor for the particular GAG residue, we use the following equation
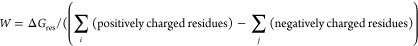
where *W* is the factor, Δ*G*_res_ is the energy obtained from per-residue energy decomposition from MM/GBSA analysis, and ∑*_i/j_* is the sum of reciprocities of the distances between GAG residues and all of the positively/negatively charged residues of the protein.

Each positive and negative residue is taken into account if it is within the cutoff of nonbonded interactions in the corresponding MD simulation. The *W* factor for the whole complex is the mean of the *W* factors for each of the GAG residues calculated from the simulations with HP dp16, and its usage for HP dp16 energy prediction would, therefore, yield the same energies as the ones obtained from the MD simulation.

**Figure 4:**
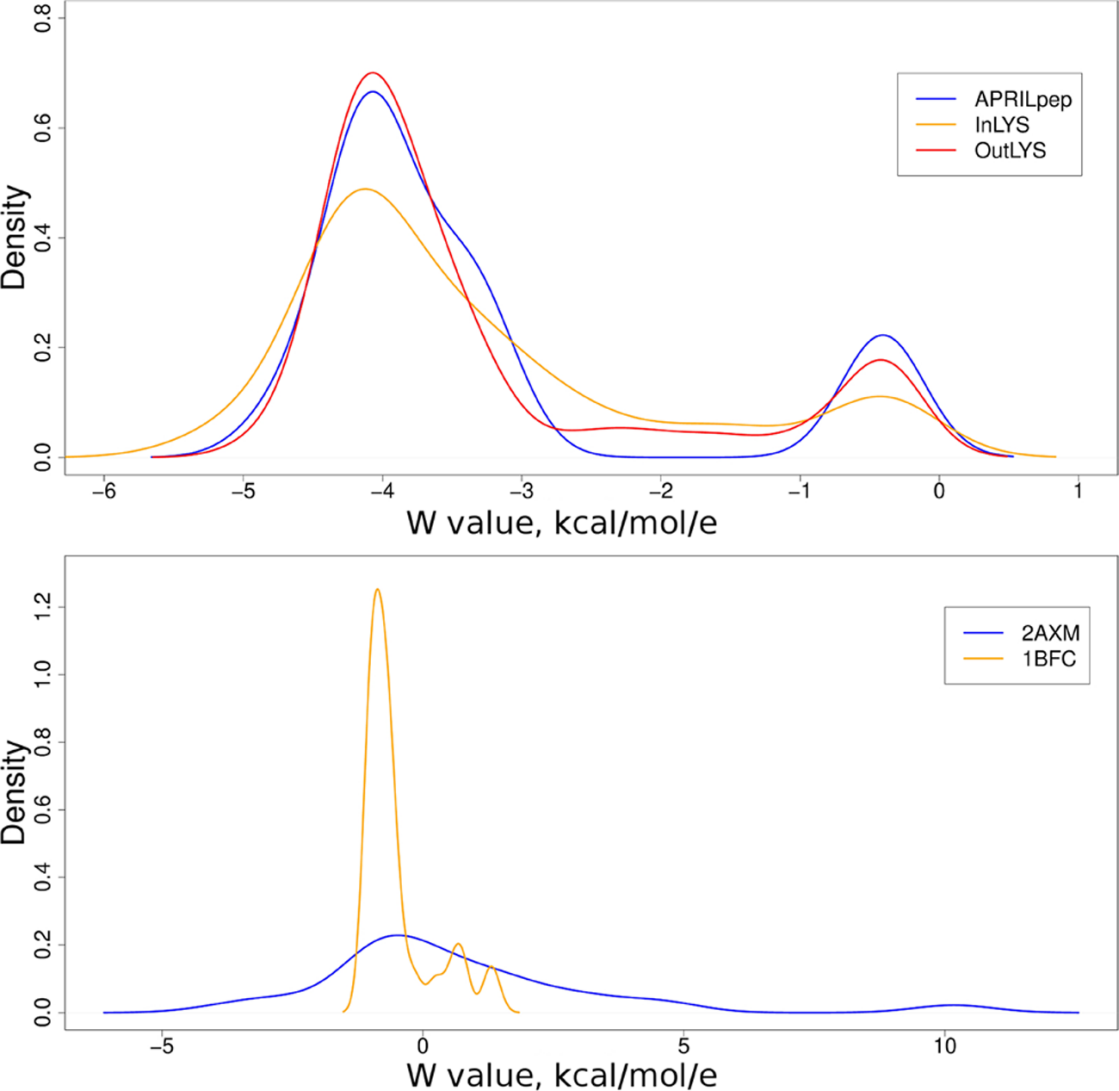
Plot of *W* value probability densities calculated from MD runs (5 MD runs for each individual complex) for HP dp16 and short peptides (top) or proteins (bottom) used in this study.

The *W* factors and their distribution (Figure 4) for the peptide–GAG complexes were very similar for the peptides: −3.35, −3.31, and −3.33 kcal mol^–1^ e^–1^ for InLys, OutLys, and N-terminal fragment of the APRIL protein, respectively. In contrast, in the case of protein complexes, they differed substantially in terms of mean of the *W* factors (0.65 and −0.50 kcal mol^–1^ e^–1^ for FGF-1 and FGF-2, respectively), and their distribution (Figure 4). It indicates that bigger and therefore more complex systems need an individual approach each time they are analyzed. However, in the case of simple and short systems (e.g., small peptide and GAG) individual approach is not necessary and the binding energy could be calculated directly using *W* factor of −3.33 kcal mol^–1^ e^–1^. In this case, performing MD simulations and binding energy analysis for longer GAG variants is not needed.

Then, similarly to the previously described procedure, the Debye–Hückel equation (Δ*G* ∼ *e*^–ϰ*r*^/*r*, where *r* is the distance and ϰ is the reversed Debye screening length) was used to calculate the *W* factor. In this approach, electrostatics screening in the electrolyte solution is taken into account. Physiological value of the ionic strength (0.15 M) was used in the calculations. The obtained data also suggested that *W* is very similar for all three peptides: 86.40, 89.13, and 85.50 kcal mol^–1^ e^–1^ for APRILpep, OutLYS, and InLys, respectively. The calculated values for the protein–GAG systems were essentially different for the two systems and compared to the peptides: −0.83 and 20.00 kcal mol^–1^ e^–1^ for 2AXM and 1BFC, respectively.

Therefore, the energies could be, in principle, predicted for HP using a specific *W* factor for each system (in the case of three peptides, *W* factors are essentially the same), and such predictions applied for longer GAGs with this particular *W* factor would yield similar values to those in the MD simulations. However, for proteins, it is not possible to make such predictions *a priori* without performing MD simulations that are needed to define the *W* factor.

Based on these results, we believe that the difference in *W* profiles for two proteins obtained by calculations based on two dissimilar physics-based models is originated in the different charge distribution topology, protein surface geometry, and thus resulting electrostatic screening effects that do not allow us to find the same uniform factor for distinct protein receptors.

### Single Pseudoatom as an Extension of the GAG Molecule

Furthermore, we aimed to design a model where only a single pseudoatom would function as an elongated lateral part of the bound GAG. Unfortunately, among the different parameters that were used, none yielded promising results in terms of reliably obtaining binding energies for the complexes compared to the ones from AA simulations, both when compared energies from MM/GBSA and LIE analysis (Table S3). Some artifacts were also observed when pseudoatom had a high negative charge (−5 or lower) causing the interruption of the MD simulation. We believe that this approach does not have broad applicability. It is rather unlikely to propose parameters for a pseudoatom that would work consistently for the complexes with different electrostatic properties and geometry topologies. Additionally, one would need to propose a complete library of parameters for pseudoatoms distinct for every different length of an elongated GAG part that pseudoatom is replacing. The possible reason for this could be that an attempt to approximate an elongated molecule with a spherical particle could probably be physically inappropriate in terms of molecular symmetry.

## Conclusions

While docking long GAG molecules may require additional laborious technical work than docking shorter (dp4/6) GAG oligomers, it is definitely worth the effort. In our approach, we use Autodock3 to find the best starting poses for the dp6 GAGs^34,41^ that can be used for further GAG elongation. At the same time, it is important to mention that our approach is not limited to any special docking software. We expect that carbohydrate- and GAG-specific docking programs as Vina-Carb^56^ or GlycoTorch Vina,^57^ respectively, which also belong to the family of Autodock programs, would perform similarly or even outperform Autodock3 for obtaining the initial structures of protein/peptide complexes with short GAGs that are to be further elongated using the procedure proposed in this manuscript. In this procedure, we elongate a docked GAG using the CG model for the monosaccharide units and use it in conventional MD simulations. In this study, it was proven that elongating GAGs substantially increases the binding energy of the complex. While it is not a linear increase of binding strength, it is still substantial when dp16 is compared to dp4 or dp6. We consider that GAG elongation using a CG model for the monosaccharide units provides nearly equivalent outcome as the AA elongation, resulting only in a 5.6% difference in assessed binding energies, without introducing excessive technical complications. This suggests that a straightforward description of electrostatic interactions of the GAG parts not establishing direct contacts with their protein target is sufficient to describe the energetics of the system accurately enough. Binding energies obtained when using our script that elongates a GAG molecule (Supporting Information) and the CG model that are provided in this work are more accurate than using shorter GAGs with a standard AA approach. This method can be utilized by any user of AMBER and standard docking software like Autodock3 in a straightforward manner. It is a great advantage that with this approach, a user can specify the length of the extended lateral part of GAG to properly satisfy his needs. We also believe that this method with minor modifications could be implemented to other linear polysaccharides or negatively charged linear polymers like nucleic acids, in general.

## Supporting Information Available

MM/PBSA free binding energy per residue decomposition analysis; DMD docking in protein–GAG systems: comparison of AA and CG GAG representations; LIE energy calculations; comparison of MM/GBSA and MM/PBSA binding free energies; RMSD of the bound HP dp16 (to investigated proteins/peptides) in AA and AA/CG representation; elongation script; script for automatic calculation of the energy of elongated GAG molecule; and frcmod file (PDF)

## Notes

All of the PDB files were downloaded from the RCSB Protein Data Bank (https://www.rcsb.org). AMBER libraries for GAG residues are accessible on the website of GAG Computational Group under “Libraries” (http://www.comp-gag.org/downloads). The following free pieces of software were used: Autodock3 for molecular docking (autodock.scripps.edu), Open Babel for formats conversion (http://openbabel.org), VMD for molecular visualization (https://www.ks.uiuc.edu/Research/vmd), and scripts for docking analysis (https://gehrcke.de/code/). All of these softwares are freely accessible on their websites. The differences between the input scripts used in molecular docking and the default values are listed on the website of GAG Computational Group under “Scripts/protocols” (http://www.comp-gag.org/downloads). All MD simulations were performed by the commercial AMBER16 molecular dynamics package (https://ambermd.org) purchased by the University of Gdańsk. The XLEAP and CPPTRAJ modules of AMBER16 were used for adding terminal residues to the polysaccharide chains and postprocessing of trajectories, respectively. MM/GBSA free binding energy calculations were performed with the MM/GBSA module of AMBER16.
