# Supplementary material for: Modeling Protein–Glycosaminoglycan Complexes: Does the Size Matter?
Source: J Chem Inf Model. 2021 Sep 8;61(9):4475–85. doi: 10.1021/acs.jcim.1c00664 (PMC8479808; doi:10.1021/acs.jcim.1c00664)
Supplement: Supplementary file 1 — ci1c00664_si_001.pdf [file ci1c00664_si_001.pdf]

Supporting Information

Modeling protein-glycosaminoglycan complexes: does the size matter?

Mateusz Marcisz[1,2], Martin Zacharias[3], Sergey A. Samsonov[1*]

[1]Faculty of Chemistry, University of Gdańsk, ul. Wita Stwosza 63, 80-308 Gdańsk, Poland.

[2]Intercollegiate Faculty of Biotechnology of UG and MUG, ul. Abrahama 58, 80-307 Gdańsk, Poland.

[3]Center of Functional Protein Assemblies, Technical University of Munich, Ernst-Otto-Fischer-Str. 8, 85748 Garching, Germany

*Corresponding Author: Sergey A. Samsonov, sergey.samsonov@ug.edu.pl

Table S1. MM/PBSA free binding energy per residue decomposition analysis for protein-GAG complexes: comparison of AA and CG GAG representations.

PDB ID	$r_{Pearson}$	$r_{Spearman}$
1GMN	0.969	0.976
1HM2	0.926	0.936
1LOH	0.734	0.883
1OFM	0.839	0.749
2D8L	0.603	0.779
2NWG	0.483	0.685
3ANK	0.931	0.944
3OGX	0.863	0.885
3OJV	0.902	0.923
All together	0.837	0.883

Table S2. DMD docking in protein-GAG systems: comparison of AA and CG GAG representations.

	1BFB		1BFC		2NWG		3C9E		2JCQ	
Parameter	AA	CG	AA	CG	AA	CG	AA	CG	AA	CG
$RMSatd_{top}$, Å	3.6	5.9	5.9	9.7	4.0	5.1	3.9	22.0	9.9	15.9
$RMSatd_{best}$, Å	2.6	4.3	2.5	5.3	2.0	3.3	3.2	5.0	5.7	6.5
$Rank_{best}$	79	34	16	70	12	11	6	71	8	69
RMSatd, Å	7.6 ± 9.6	7.5 ± 2.1	5.9 ± 3.9	8.8 ± 1.8	5.6 ± 3.3	8.0 ± 3.0	6.9 ± 3.0	13.2 ± 5.2	11.2 ± 4.5	14.6 ± 4.9
$RMSatd_{top\ cluster}$, Å	3.2 ± 0.4	7.5 ± 0.7	3.5 ± 0.5	7.5 ± 0.4	3.6 ± 0.7	5.9 ± 0.6	5.8 ± 1.6	8.1±0.3	10.1 ± 1.7	15.9 ± 1.1
$r(\Delta G_{total}$~RMSD)	0.29	0.36	0.29	0.18	0.54	0.44	0.29	-0.42	0.23	0.16
$r(\Delta G_{elect}$~RMSD)	0.46	0.47	0.62	0.21	0.75	0.54	0.66	-0.08	-0.75	0.52
Number of correctly predicted residues	9 of 10	8 of 10	8 of 10	8 of 10	7 of 10	5 of 10	6 of 10	2 of 10	1 of 7	2 of 5
Number of correctly charged predicted residues	6	6	7	6	7	5	4	2	1	2
Number of correctly predicted uncharged polar residues	2	2	1	2	0	0	2	0	0	0

$RMSatd_{top}$: structural difference between the best scored docked structure and the corresponding experimental structure; $RMSatd_{best}$: structural difference between the docked structure which is most similar structure to the corresponding experimental structure and the corresponding experimental structure; $Rank_{best}$; rank of the docked structure which is most similar structure to the corresponding experimental structure; RMSatd: mean structural difference between all docked structures and the corresponding experimental structure; $RMSatd_{top\ cluster}$: mean structural difference between all docked structures from the cluster of solutions with the highest scores and the corresponding experimental structure; $r(\Delta G_{total}$~RMSatd): Pearson correlation coefficient for total free binding energy obtained by MM/PBSA and RMSatd of all docked structures; $r(\Delta G_{elect}$~RMSatd): Pearson correlation coefficient for *in vacuo* electrostatic free binding energy component and RMSatd of all docked structures; number of correctly predicted residues, number of correctly charged predicted residues, number of correctly predicted uncharged polar residues were referenced to the 10 protein residues with the highest impacts on binding according to the per residue decomposition for the corresponding X-ray structures.
The data for AA GAG representation are taken from the original work on the DMD approach [Samsonov, Gehrcke, Pisabarro 2014 JCIM]

Table S3. LIE analysis.

As a complement to MM/GBSA analysis we performed LIE energy calculations with CPPTRAJ scripts. In case of this approach we used standard parameters with dielectric constant of 80. This method resulted in much worse energy estimations.[Table3] This is probably due to the fact that LIE analysis to be used at it's full potential requires calibration with experimental results. Additionally, LIE method was not developed to work with mixed AA/CG model as our model with pseudo-atoms. Therefore we think that this method is not suited as proper energy assessment tool for our GAG model.

	AA simulations	AA/CG simulations	
	Only AA residues	Mixed model 1*	Mixed model 2**
2AXM	-39.9	-53.4	-39.2
1BFC	-50.1	-57.0	-58.9
April peptide	-34.6	-40.7	-38.2
InLYS	-18.4	-33.6	-21.8
OutLYS	-22.5	-27.7	-28.0

*Elongated fragments of the GAG replaced with CG residues.

**AA Residues replaced with CG residues based on energy decomposition

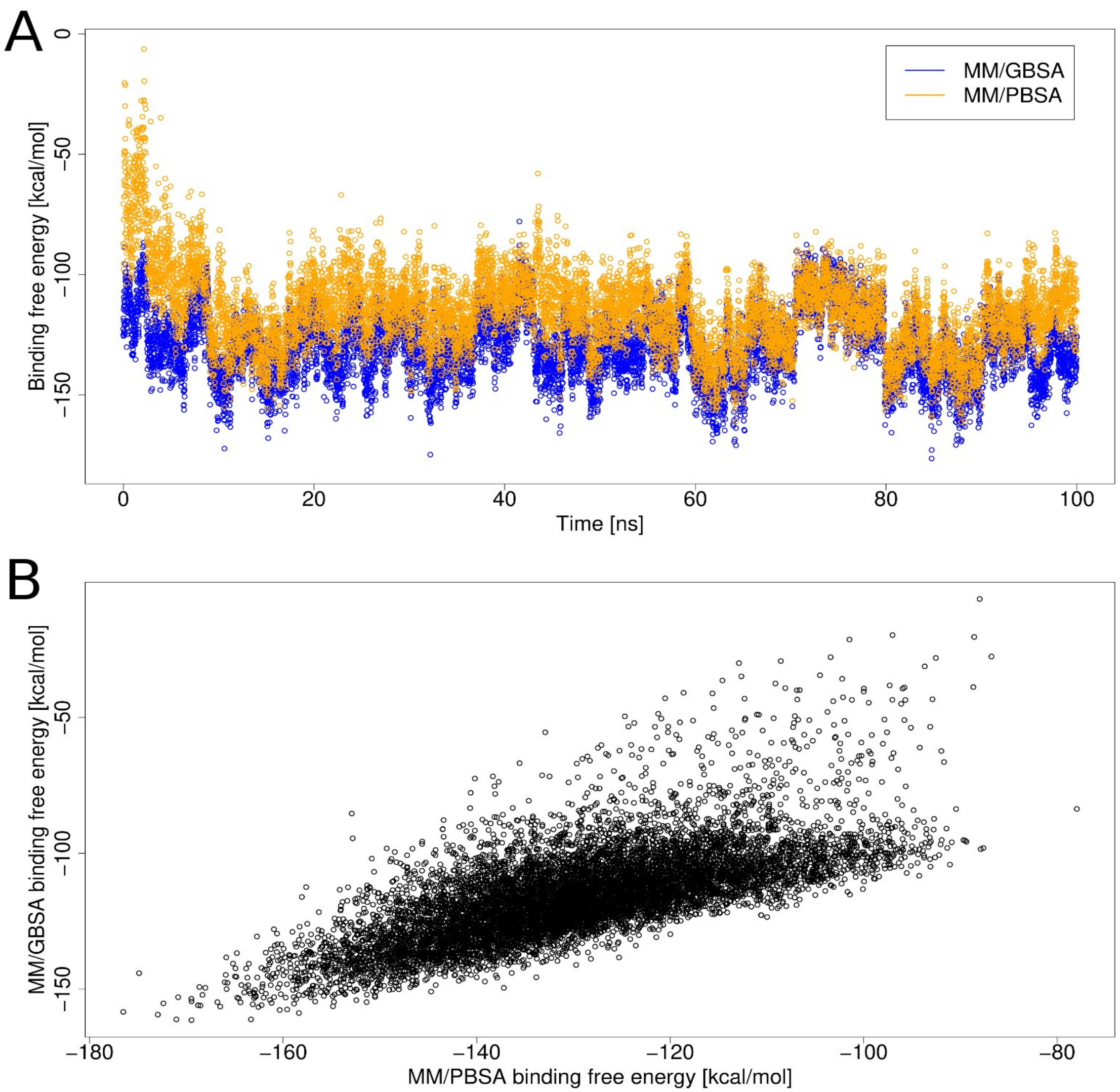

Figure S1. Comparison of MM-GBSA and MM-PBSA binding free energies obtained for all frames in an MD simulation for 1BFC. A. Binding free energies in the course of the MD simulation; 2. Correlation between binding free energies obtained by two methods (Pearson correlation coefficient for this example is 0.70 and is 0.73 when only last 90 ns are analyzed).

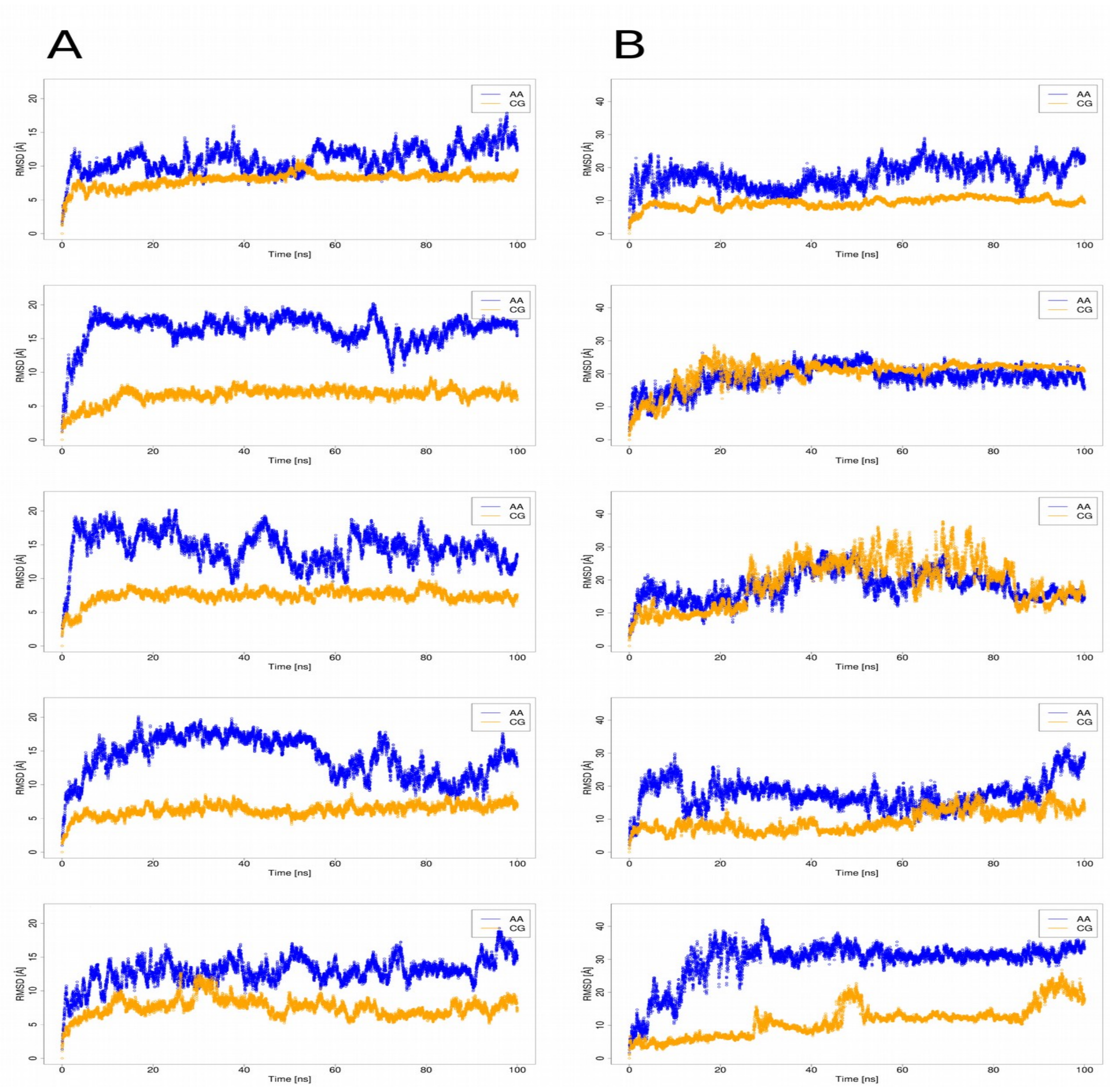

Figure S2. RMSD of the bound HP dp16 in AA and AA/CG representations in 5 MD simulations for complexes with: A. 1BFC and B. APRIL peptide.

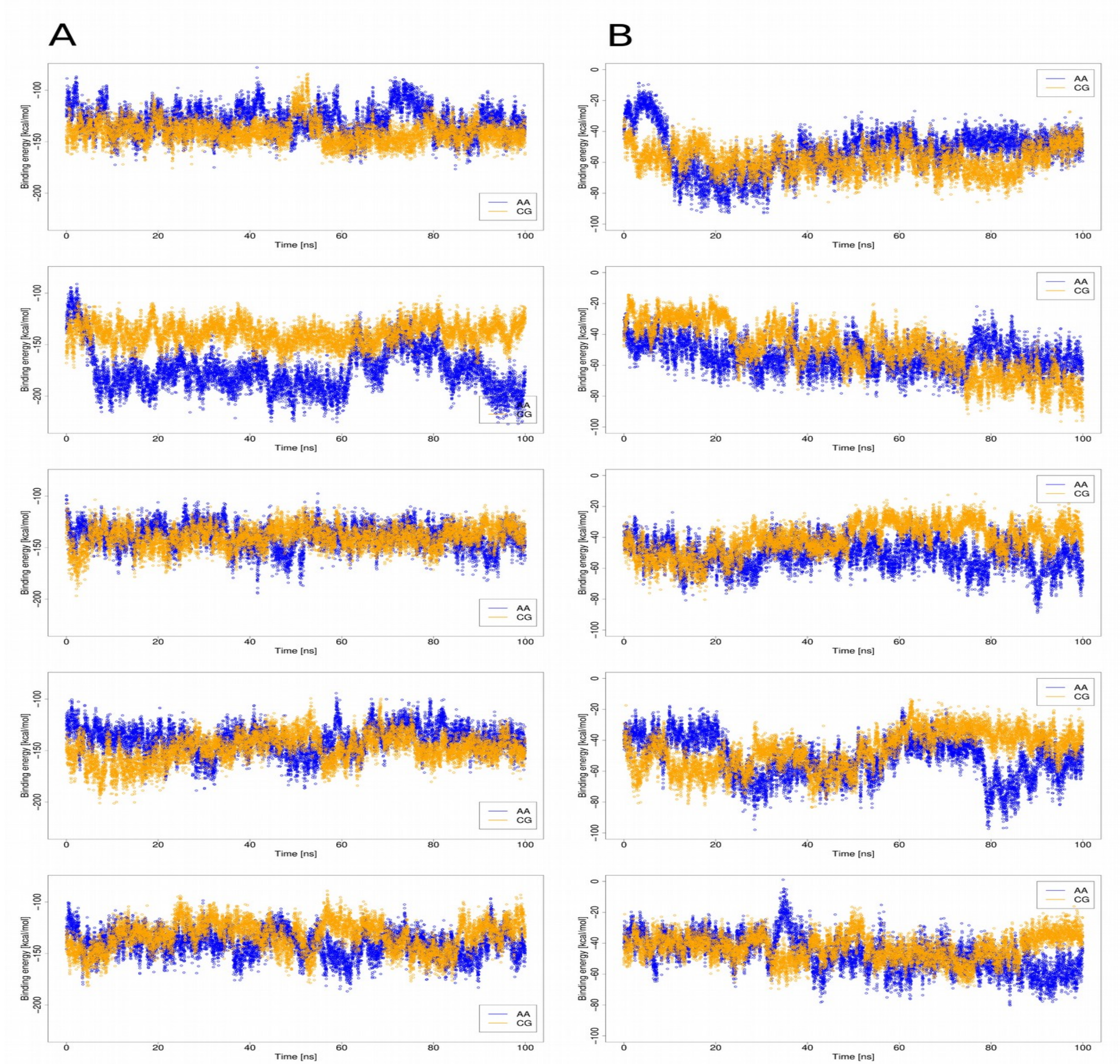

Figure S3. MM-GBSA free binding energy of the bound HP dp16 in AA and AA/CG representations in 5 MD simulations for complexes with: A. 1BFC and B. APRIL peptide.

Elongation Script:

Here we provide a script that automatically elongates GAG molecule in the chosen directions.

```
#ROH end

c=`cat gag.pdb | awk '{print $5}' | uniq | head -2 | tail -1`

a=$((c-1))


awk -v c=${c} '{if ($5==c) print $0 }' gag.pdb  | grep 'C1\|C2\|C3\|C4\|\C5\|O5\s ' > ring_start
awk -v a=${a} '{if ($5==a) print $0 }' gag.pdb  | grep 'C1\|C2\|C3\|C4\|\C5\|O5\s ' > ring_end


#creating points corresponding to a GAG: beware of the direction
echo "How many residues to add to the start of the GAG?"
read n

x1=`awk '{ sum += $6; n++ } END { if (n > 0) print sum / n; }' ring_start`
y1=`awk '{ sum += $7; n++ } END { if (n > 0) print sum / n; }' ring_start`
z1=`awk '{ sum += $8; n++ } END { if (n > 0) print sum / n; }' ring_start`
x2=`awk '{ sum += $6; n++ } END { if (n > 0) print sum / n; }' ring_end`
y2=`awk '{ sum += $7; n++ } END { if (n > 0) print sum / n; }' ring_end`
z2=`awk '{ sum += $8; n++ } END { if (n > 0) print sum / n; }' ring_end`

vector=`echo $x1 $y1 $z1 $x2 $y2 $z2 | awk '{print $4-$1,$5-$2,$6-$3}'`
dx=`echo $vector |  awk '{print $1}'`
dy=`echo $vector |  awk '{print $2}'`
dz=`echo $vector |  awk '{print $3}'`

rm output.xyz

for (( i=1; i<=n; i++))
```

```
do
echo $x2 $y2 $z2 | awk -v dx=${dx} -v dy=${dy} -v dz=${dz} -v i=${i} '{printf "%7.3f  %7.3f  %7.3f\n",$1+dx*i, $2+dy*i, $3+dz*i}'
>> output.xyz
done

./xyz_to_pdb output.xyz output.pdb

cat output.pdb | grep '^ATOM' > 1
mv 1 output_start.pdb


#second end


d=`cat gag.pdb | awk '{print $5}' | uniq | tail -2`

e=$((d-1))


awk -v e=${e} '{if ($5==e) print $0 }' gag.pdb  | grep 'C1\|C2\|C3\|C4\|\C5\|O5\s ' > ring_start
awk -v d=${d} '{if ($5==d) print $0 }' gag.pdb  | grep 'C1\|C2\|C3\|C4\|\C5\|O5\s ' > ring_end


#creating points corresponding to a GAG: beware of the direction
echo "How many residues to add to the end of the GAG?"
read n

x1=`awk '{ sum += $6; n++ } END { if (n > 0) print sum / n; }' ring_start`
y1=`awk '{ sum += $7; n++ } END { if (n > 0) print sum / n; }' ring_start`
z1=`awk '{ sum += $8; n++ } END { if (n > 0) print sum / n; }' ring_start`
x2=`awk '{ sum += $6; n++ } END { if (n > 0) print sum / n; }' ring_end`
y2=`awk '{ sum += $7; n++ } END { if (n > 0) print sum / n; }' ring_end`
z2=`awk '{ sum += $8; n++ } END { if (n > 0) print sum / n; }' ring_end`
```

```
vector=`echo $x1 $y1 $z1 $x2 $y2 $z2 | awk '{print $4-$1,$5-$2,$6-$3}'`
dx=`echo $vector |  awk '{print $1}'`
dy=`echo $vector |  awk '{print $2}'`
dz=`echo $vector |  awk '{print $3}'`

rm output.xyz

for (( i=1; i<=n; i++))
do
echo $x2 $y2 $z2 | awk -v dx=${dx} -v dy=${dy} -v dz=${dz} -v i=${i} '{printf "%7.3f  %7.3f  %7.3f\n",$1+dx*i, $2+dy*i, $3+dz*i}'
>> output.xyz
done

./xyz_to_pdb output.xyz output.pdb

cat output.pdb | grep '^ATOM' > 1
mv 1 output_end.pdb

rm modified
cp output_start.pdb test.pdb
n=`wc -l test.pdb | awk '{print $1}'`
for  ((i = 1; i <= $n; i++))
do
echo $i
if [ $i   -le    9 ]
then
tail -$i test.pdb | head -1 | sed  "s/H            0/H            $i/" >> modified
elif [ $i   -le    99 ]
then
tail -$i test.pdb | head -1 | sed  "s/H            0/H           $i/" >> modified
fi
if [ $i   -gt    99 ]
then
tail -$i test.pdb | head -1 | sed  "s/H            0/H          $i/" >> modified
```

```
fi
done
mv modified modified1.pdb


cp output_end.pdb test.pdb
n=`wc -l test.pdb | awk '{print $1}'`
for  ((i = 1; i <= $n; i++))
do
echo $i
if [ $i   -le    9 ]
then
head -$i test.pdb | tail -1 | sed  "s/H           0/H           $i/" >> modified
elif [ $i   -le    99 ]
then
head -$i test.pdb | tail -1 | sed  "s/H           0/H          $i/" >> modified
fi
if [ $i   -gt    99 ]
then
head -$i test.pdb | tail -1 | sed  "s/H           0/H         $i/" >> modified
fi
done
mv modified modified2.pdb



cat modified1.pdb gag.pdb modified2.pdb > 1.pdb

sed 's/TER//g' 1.pdb > 2.pdb
sed 's/END//g' 2.pdb > new_gag.pdb

rm 1.pdb 2.pdb
```

Energy calculating script:

Here we provide a script that automatically calculates energy of elongated GAG molecule in the chosen direction.

```
#Create files from gag.pdb and protein.pdb
#gags: from full_gag.pdb
rm ring*
echo "Which residue?"
read a
echo "Which direction: -1-from the beginning; 1: to the end"
read b
echo $a, $b
c=$((a+b))
echo $c

awk -v a=${a} '{if ($5==a) print $0 }' full_gag.pdb  | grep 'C1\|C2\|C3\|C4\|\C5\|O5\s ' > ring_start
awk -v c=${c} '{if ($5==c) print $0 }' full_gag.pdb  | grep 'C1\|C2\|C3\|C4\|\C5\|O5\s ' > ring_end


#protein
rm charged_points.pdb
numbers=`awk '{if ($5!="") print $5}' protein.pdb | uniq | tail -1`

awk '{if (($3=="N") && ($5==1)) print $0}' protein.pdb > charged_points.pdb

for ((i=1; i<=$numbers; i++))
do
awk -v i=${i} '{if (($5==i) && ($3=="NZ")) print $0}' protein.pdb | grep LYS >> charged_points.pdb
awk -v i=${i} '{if (($5==i) && ($3=="CZ")) print $0}' protein.pdb | grep ARG >> charged_points.pdb
awk -v i=${i} '{if (($5==i) && ($3=="CG")) print $0}' protein.pdb | grep ASP >> charged_points.pdb
awk -v i=${i} '{if (($5==i) && ($3=="CD")) print $0}' protein.pdb | grep GLU >> charged_points.pdb
done
```

```
awk -v numbers=${numbers} '{if (($3=="C") && ($5==numbers)) print $0}' protein.pdb >> charged_points.pdb

#creating points corresponding to a GAG: beware of the direction and numbers
echo "How many to add?"
read n

x1=`awk '{ sum += $6; n++ } END { if (n > 0) print sum / n; }' ring_start`
y1=`awk '{ sum += $7; n++ } END { if (n > 0) print sum / n; }' ring_start`
z1=`awk '{ sum += $8; n++ } END { if (n > 0) print sum / n; }' ring_start`
x2=`awk '{ sum += $6; n++ } END { if (n > 0) print sum / n; }' ring_end`
y2=`awk '{ sum += $7; n++ } END { if (n > 0) print sum / n; }' ring_end`
z2=`awk '{ sum += $8; n++ } END { if (n > 0) print sum / n; }' ring_end`

vector=`echo $x1 $y1 $z1 $x2 $y2 $z2 | awk '{print $4-$1,$5-$2,$6-$3}'`
dx=`echo $vector |  awk '{print $1}'`
dy=`echo $vector |  awk '{print $2}'`
dz=`echo $vector |  awk '{print $3}'`

rm output.xyz

for (( i=1; i<=n; i++))
do
echo $x2 $y2 $z2 | awk -v dx=${dx} -v dy=${dy} -v dz=${dz} -v i=${i} '{printf "%7.3f  %7.3f  %7.3f\n",$1+dx*i, $2+dy*i, $3+dz*i}' >> output.xyz
done

./xyz_to_pdb output.xyz output.pdb

cat output.pdb | grep '^ATOM' > 1
mv 1 output.pdb

#Calculate energies
#n: number of residues added
#m: number of charged centers/points
n=`wc -l output.pdb | awk '{print $1}'`
```

```
m=`wc -l charged_points.pdb | awk '{print $1}'`
echo $n $m

rm -R Data
mkdir Data
for ((i=1; i<=n; i++))
do
for ((j=1; j<=m; j++))
do
gag=`head -"$i" output.pdb | tail -1`
protein=`head -"$j" charged_points.pdb | tail -1`
echo $gag $protein
echo $i
echo $gag $protein | awk '{if (($12 ~ /N/) || ($12=="CZ")) print (($5-$15)^2+($6-$16)^2+($7-$17)^2)^(1/2); else print -(($5-$15)^2+($6-$16)^2+($7-$17)^2)^(1/2)}' >> Data/"$i".out
done
done

rm final_energies

#Change the W value (-4.06 here) for your calcuclted value.
for ((i=1; i<=n; i++))
do
awk -v i=${i} '{ sum += -4.06/$1; n++ } END { if (n > 0) print i, sum; }' Data/"$i".out >> final_energies
done
```

#Atoms for point charge approximation of GAG monomeric units

MASS
Z1 225.00 Half of the HP dp2 mass

BOND
Z1-Z1 120 5.2 Description
Z1-Cg 120 5.2
Os-Z1 120 2.8

ANGL
Z1-Z1-Z1 100.0 160.0 Description
Z1-Z1-Cg 100.0 160.0
Z1-Cg-H2 70.0 108.5
Z1-Cg-Cg 70.0 108.5
Z1-Cg-Os 60.0 110.0
Cg-Os-Z1 100.0 160.0
Os-Z1-Z1 100.0 160.0

DIHE
Z1-Z1-Z1-Z1 1 1.00 0.0 1. Description
Z1-Z1-Z1-Cg 1 1.00 0.0 1.
Z1-Z1-Cg-Cg 1 0.16 0.0 3.
Z1-Cg-Cg-H1 1 0.16 0.0 3.
Z1-Cg-Cg-H2 1 0.16 0.0 3.
Z1-Z1-Cg-H2 1 0.16 0.0 3.
Z1-Z1-Cg-Os 1 0.16 0.0 3.
Z1-Cg-Cg-Ng 1 -1.30 0.0 1.
Z1-Cg-Cg-Cg 1 -0.27 0.0 1.
Z1-Cg-Os-Cg 1 -0.27 0.0 1.
Cg-Cg-Os-Z1 1 0.16 0.0 3.
Cg-Os-Z1-Z1 1 0.16 0.0 3.

H1-Cg-Os-Z1	1	0.27	0.0	3.
Z1-Cg-Cg-Os	1	0.16	0.0	3.
Os-Z1-Z1-Z1	1	0.16	0.0	3.

NONBON

Z1	4.0000	3.4000	Similar to ring CG particles from the 2015
